# Long noncoding RNAs in the progression, metastasis, and prognosis of osteosarcoma

**DOI:** 10.1038/cddis.2016.272

**Published:** 2016-09-29

**Authors:** Zuozhang Yang, Xiaojuan Li, Yihao Yang, Zewei He, Xin Qu, Ya Zhang

**Affiliations:** 1 Bone and Soft Tissue Tumors Research Center of Yunnan Province, Department of Orthopaedics, The Third Affiliated Hospital of Kunming Medical University (Tumor Hospital of Yunnan Province), Kunming 650118, China

## Abstract

Long noncoding RNAs (lncRNAs) are a class of non-protein-coding molecules longer than 200 nucleotides that are involved in the development and progression of many types of tumors. Numerous lncRNAs regulate cell proliferation, metastasis, and chemotherapeutic drug resistance. Osteosarcoma is one of the main bone tumor subtypes that poses a serious threat to adolescent health. We summarized how lncRNAs regulate osteosarcoma progression, invasion, and drug resistance, as well as how lncRNAs can function as biomarkers or independent prognostic indicators with respect to osteosarcoma therapy.

## Facts

- Long noncoding RNAs (lncRNAs) regulate cell proliferation in osteosarcoma.
- LncRNAs regulate cell invasion and chemotherapeutic drug resistance in human osteosarcoma.
- LncRNAs function as biomarkers and independent prognostic indicators with respect to osteosarcoma therapy.

## Open Questions

- How do lncRNAs regulate osteosarcoma progression and invasion?
- How do lncRNAs regulate osteosarcoma chemotherapeutic drug resistance?
- Can lncRNAs be used as biomarkers or prognostic indicators with respect to human osteosarcoma treatment?

Osteosarcoma is the most common malignant bone tumor in children and adolescents. It is a genetically unstable and highly malignant mesenchymal tumor of bone characterized by structural chromosomal alterations.^1, 2^ Malignant osteosarcoma cells produce osteoid matrix and fibrillary stroma.^3^ The most common osteosarcoma subtypes are osteoblastic osteosarcoma, chondroblastic osteosarcoma, and fibroblastic sarcoma.^4, 5^ Osteosarcoma occurs predominantly in adolescents and young adults and accounts for ~5% of childhood cancers. Most osteosarcoma patients are diagnosed under the age of 25 years, and the disease occurs more often in males than in females.^6^ Osteosarcoma often exhibits locally invasive growth. Pulmonary metastases are often seen in patients with aggressive tumors. Both biopsy findings and classic X-ray findings contribute to the diagnosis of osteosarcoma and yield important information that can be used to select appropriate therapies.^7, 8^ In most osteosarcoma patients, chemotherapy and/or radiation therapy are usually administered before or after surgery to prevent tumors from spreading throughout the body. However, patients with distant metastases still fare poorly, as the 5-year survival rate in these patients is ~20%.^9, 10^ Thus, developing comprehensive and multidimensional treatments for osteosarcoma is necessary, and gene therapies using viral vectors, immune therapies, antiangiogenic therapies, and proapoptotic therapies have been investigated regarding their application in patients with osteosarcoma.

To date, the molecular mechanism underlying osteosarcoma development remains unclear. The majority of previous studies have focused on protein-coding genes as crucial components involved in the progression and metastasis of osteosarcoma and have overlooked the vast landscape of noncoding genes.

Since the invention of DNA sequencing methods and the completion of the draft human genome sequence, researchers have found that only 1.5% of 3.2 billion nucleotide pairs code for proteins and that the other 98.5% of DNA sequences do not code for proteins. These sequences are recognized as junk sequences that have accumulated because of the process of evolution.^11, 12^ ENCODE (Encyclopedia of DNA Elements) projects postulate that 80% of genome sequences are transcribed into primary transcripts and have biochemical functions.^13, 14^ The concept of ‘junk DNA' has rapidly attracted the attention of researchers. According to their biological functions, noncoding RNAs can be divided into housekeeping noncoding RNAs and regulatory noncoding RNAs.^15, 16, 17^ Housekeeping noncoding RNAs comprise ribosomal RNAs,^18, 19, 20^ transfer RNAs,^21, 22^ small nuclear RNAs,^23, 24, 25^ small nucleolar RNAs,^26, 27, 28^ guide RNAs,^29, 30, 31, 32^ and telomerase RNAs.^33, 34^ Regulatory noncoding RNAs comprise small interfering RNAs (siRNAs),^35, 36, 37^ micro RNAs (microRNA),^38, 39, 40^ piwi-interacting RNAs,^41, 42, 43^ and long noncoding RNAs (lncRNAs).^44, 45^

**Figure 1:**
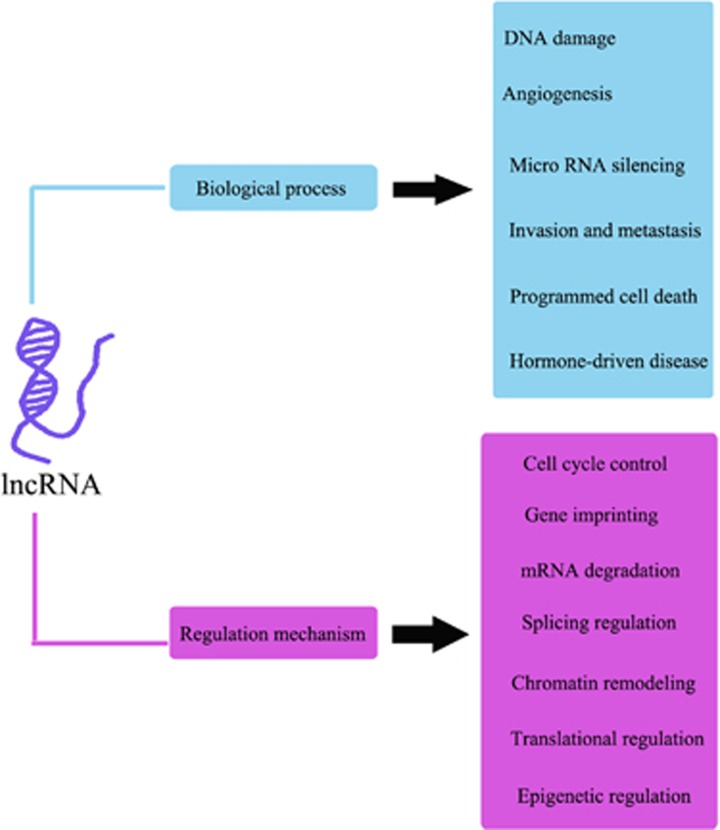
Biological processes are regulated by lncRNAs, and several regulatory mechanisms are shown

lncRNAs are a large and diverse class of non-protein-coding transcripts longer than 200 nucleotides.^46, 47^ lncRNAs have recently gained widespread attention and have been shown to have crucial roles in various biological regulatory processes. lncRNA sequences are conserved, and lncRNA expression profiles in adult tissues are broad. lncRNA messenger RNAs (mRNAs) are generally less abundant than protein-coding mRNAs but exhibit stronger tissue- and cell-specific lncRNA expression patterns.^48, 49^ Most lncRNAs are transcribed by RNA polymerase II enzymes that lack open-reading frames and are expressed in specific tissues and/or during specific developmental stages, demonstrating that the genes encoding these molecules are strictly regulated with respect to tissue development. Previous research has mainly focused on microRNAs and siRNAs. lncRNAs have recently been found to be involved in development, differentiation, and proliferation, as well as cell cycle regulation and programmed cell death.^50, 51, 52, 53^ They also have important roles in the progression and metastasis of various tumors, such as colon cancer, liver cancer, breast cancer, bladder cancer, and cervical cancer^51, 54, 55, 56, 57, 58, 59^ (Figure 1). In this paper, we reviewed the biological functions of lncRNAs and the molecular mechanisms underlying these functions with respect to osteosarcoma progression. Chemotherapy drug resistance remains an obstacle affecting osteosarcoma treatment. We therefore also summarized the lncRNAs that are correlated with chemotherapeutic drug resistance in osteosarcoma therapy. Furthermore, we summarized several lncRNAs that can function as independent prognostic indicators of overall survival and can serve as useful biomarkers of osteosarcoma progression and prognosis. An overview of the lncRNAs that are associated with osteosarcoma is shown in Table 1.

## LncRNA Regulates Signaling Pathways in Osteosarcoma

Developing effective and targeted therapies for osteosarcoma is dependent on gaining an improved understanding of the molecular mechanisms underlying osteosarcomagenesis, proliferation, invasion, and metastasis.^60^ To date, the molecular mechanism underlying osteosarcoma development has not been elucidated. It is known that Wnt signaling is involved in osteosarcoma development, metastasis, and drug resistance. For example, inhibiting Wnt signaling by targeting c-Met, a Wnt-regulated proto-oncogene, was shown to be useful for treating osteosarcoma, suggesting that the Wnt signaling pathway is involved in osteosarcoma development and metastasis.^61^ Chemotherapeutic drug resistance represents a major obstacle with respect to osteosarcoma treatment, due in part to phenotypic cell transitions toward stem-like phenotypes caused by exposure to conventional chemotherapeutics.^62, 63^ However, the combination of a Wnt/*β*-catenin signaling pathway inhibitor and doxorubicin prevented the upregulation of factors linked to these types of transitions and was thus envisaged as a means of overcoming adaptive resistance.^64^ Aberrant hedgehog (Hh) signaling pathway activity has been observed in osteosarcoma cell lines, as well as in primary human osteosarcoma tissue specimens, and exerts promigratory effects leading to the development of osteoblastic osteosarcoma.^65^ Other studies have also demonstrated that dysregulated Hh signaling contributes to poor clinical outcomes in osteosarcoma therapy.^66, 67^ Bone morphogenetic protein (BMP) signaling pathways have been reported to induce mesenchymal stem cell osteogenic commitments and terminal differentiation, which is initiated by BMP ligand heterodimer (BMPR I and II) binding and signal transduction through the Smad pathway, as well as mitogen-activated protein kinase (MAPK) phosphorylation.^68, 69, 70, 71^ In particular, of the 31 different types of known BMP ligands, BMP-2, -4, -6, -7, and -9 have significant roles in osteogenesis induction in osteosarcoma.^72, 73, 74^ Moreover, the Notch pathway has been described as an oncogene that is involved in osteosarcoma proliferation, migration, invasiveness, and oxidative stress resistance, as well as the expression of markers associated with stemness or tumor-initiating cells.^75, 76, 77, 78^ Moreover, this pathway has a vital role in regulating tumor angiogenesis and vasculogenesis in osteosarcoma.^79^ The phosphatidylinositol 3-kinase (PI3K)/Akt pathway is also thought to be one of the most important oncogenic pathways in human osteosarcoma.^80, 81^ A large number of regulatory factors regulate osteosarcoma cell proliferation, apoptosis, angiogenesis, metastasis, and chemotherapy drug sensitivity by regulating PI3K/Akt signaling, including p53,^82, 83^ VEGF,^84^ CXCR7,^85^ Aurora-B,^86^ microRNA-221,^87^ cyclooxygenase-2,^88^ BYL719, a PI3K inhibitor,^89^ and LY294002.^90^ All these signaling pathways are interconnected to regulate osteosarcoma progression and migration.

**Figure 2:**
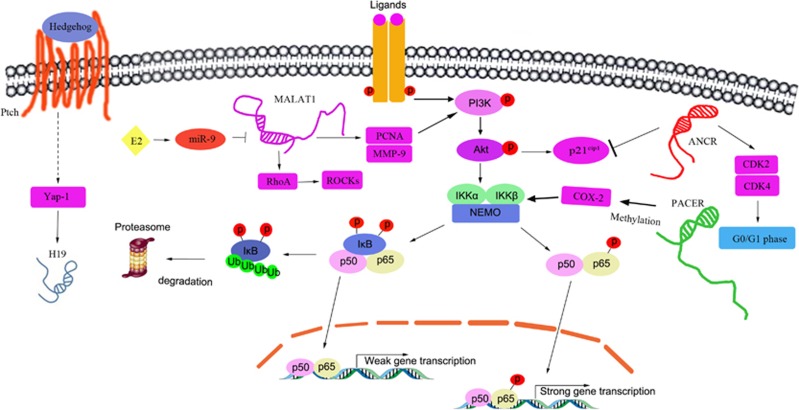
Osteosarcoma cell proliferation is regulated by lncRNAs, including H19, MALAT1, ANCR, and PACER. These osteosarcoma-related lncRNAs are involved in the PI3K/Akt signaling pathway, NF-*κ*B signaling pathway, and Hh/Yap1 signaling pathway

To date, few studies have reported the roles of lncRNAs in osteosarcoma osteogenesis, development, invasion, metastasis or chemotherapy resistance. Alterations in the expression of several lncRNAs have been observed in osteosarcoma. Li *et al.*^91^ detected the expression profiles of numerous lncRNAs via microarray analysis and observed several differentially expressed lncRNAs in osteosarcoma tissues compared with paired adjacent noncancerous tissues. In particular, 25 733 lncRNAs were expressed in osteosarcoma, including 403 consistently over-regulated lncRNAs involved in 34 pathways and 798 consistently under-regulated lncRNAs involved in 32 pathways, across all samples (2.0-fold, *P*<0.05), suggesting that lncRNAs can function as therapeutic targets and serve as novel candidate biomarkers with respect to osteosarcoma diagnosis and prognosis.^91^ P50-associated COX-2 extragenic RNA (PACER) was overexpressed in clinical osteosarcoma tissues and cell lines influenced by DNA methylation, activated the *COX-2* gene in an NF-*κ*B-dependent manner and functioned as an oncogene in osteosarcoma.^92^ Metastasis-associated lung adenocarcinoma transcript 1 (MALAT1), one of the first cancer-associated lncRNAs to be identified, is expressed in numerous tissues, is highly abundant in neurons and is involved in regulating the recruitment of SR family pre-mRNA-splicing factors to sites of transcription involved not only in nuclear processes but also in synapse function.^93^ Aberrant MALAT1 expression has been observed in many types of tumors, including hepatocellular carcinoma, cervical cancer, breast cancer, ovarian cancer, and colorectal cancer. Dong *et al.*^94^ found that MALAT1 was highly expressed in human osteosarcoma tissues and that its expression level was closely correlated with pulmonary metastasis. Moreover, they found that MALAT1 knockdown suppressed human osteosarcoma cell proliferation, invasion, and metastasis *in vitro* and *in vivo*. They also explored the molecular mechanisms underlying the function of MALAT1 in osteosarcoma and observed that MALAT1 inhibited tumor growth and metastasis via the PI3K/AKT signaling pathway, as the expression levels of proliferating cell nuclear antigen, matrix metallopeptidase 9 (MMP-9), phosphorylated PI3Kp85*α*, and Akt were significantly decreased in MALAT1-knockdown cells.^94^ Cai *et al.*^95^ observed similar results in MALAT1 siRNA-treated osteosarcoma cells. They showed that MALAT1 knockdown inhibited osteosarcoma cell proliferation and migration, induced osteosarcoma cell cycle arrest and cell apoptosis, and delayed tumor growth in an osteosarcoma xenograft model. Specifically, they found that MALAT1 siRNA administration decreased the protein expression levels of RhoA and its downstream effectors, the Rho-associated coiled-coil containing protein kinases (ROCKs). Consistent with these studies, high-dose 17*β*-estradiol (E2) treatment markedly downregulated MALAT1-mediated osteosarcoma cell proliferation, migration, invasion, and metastasis by upregulating miR-9 in E2-dose-dependent and ER-independent manners. In addition, MALAT1 downregulation promoted the formation of the SFPQ/PTBP2 complex.^96^ Moreover, Taniguchi *et al.*^97^ found that MALAT1 contains a theoretical Myc-6-target sequence that includes an E-box-like motif (at positions −258 to −251). Interestingly, knockdown of the putative Myc-6 target MALAT1 obviously impaired MG63 cell growth. In general, Myc-6 appears to exert its tumor-suppressive effects, at least in part, through the specific downregulation of MALAT1. The Hh signaling pathway hass important roles in vertebrate embryonic development and growth regulation, functions as a morphogen and mitogen, and is normally deactivated after embryogenesis. However, Hh signaling is reactivated and upregulated in various cancers, including osteosarcoma, resulting in high levels of yes-associated protein 1 (Yap1) expression. Yap1, a potent oncogene expressed in both human and mouse tumor tissues, is amplified in various cancers. Hh signaling inhibition reduces Yap1 expression, and Yap1 knockdown significantly inhibits tumor progression. Chan *et al.*^98^ found that aberrant Hh signaling in mature osteoblasts is responsible for the pathogenesis of osteoblastic osteosarcoma. Moreover, Hh signaling upregulation and Yap1 overexpression lead to aberrant lncRNA H19 expression in malignant osteosarcoma. The lncRNAs involved in osteosarcoma and osteosarcoma-related signaling pathways are shown in Figure 2.

## LncRNA Regulates Osteosarcoma Metastasis

Distant metastases are commonly observed in patients with osteosarcoma after surgery. It is estimated that metastases have been found in 85% of patients with osteosarcoma. The most common site of osteosarcoma metastasis is the lung. Metastatic osteosarcoma is difficult to control, and respiratory symptoms appear only in the setting of extensive involvement. Osteosarcoma also metastasizes to other bone and soft tissue locations. This issue is still controversial, as some authors have argued that bone metastases may actually be multifocal osteosarcomas rather than actual metastases. Death from osteosarcoma is usually a result of pulmonary metastasis and respiratory failure because of widespread progression.

**Figure 3:**
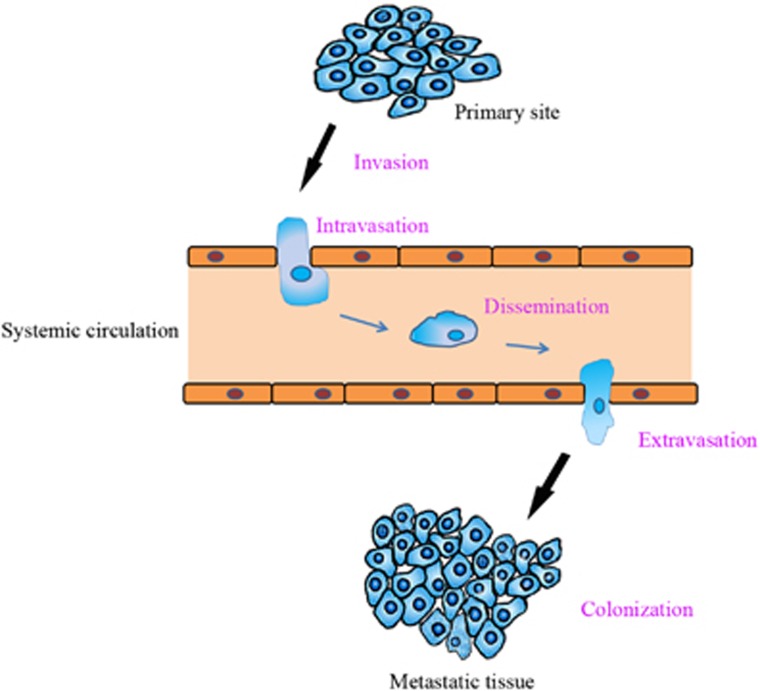
Tumor invasion and metastasis is a multilink, multistep complex process. Tumor cells at primary tumor sites invade surrounding tissues, migrate through the blood or lymph and localize in distal targeted tissues. This process is divided into the following five stages: invasion, intravasation, dissemination, extravasation, and colonization

Tumor invasion and metastasis is a multilink, multistep complex process comprising invasion, intravasation, dissemination, extravasation, and colonization. Briefly, tumor cells alter cell–extracellular matrix (ECM) interactions at the primary tumor site, escape from the primary site and invade adjacent tissues, and translocate through the vasculature to migrate to other systems. Then, these metastatic cancer cells anchor to distant vessel walls and extravasate into their destination tissues (Figure 3) before finally proliferating from microscopic growths to form secondary tumors.

**Figure 4:**
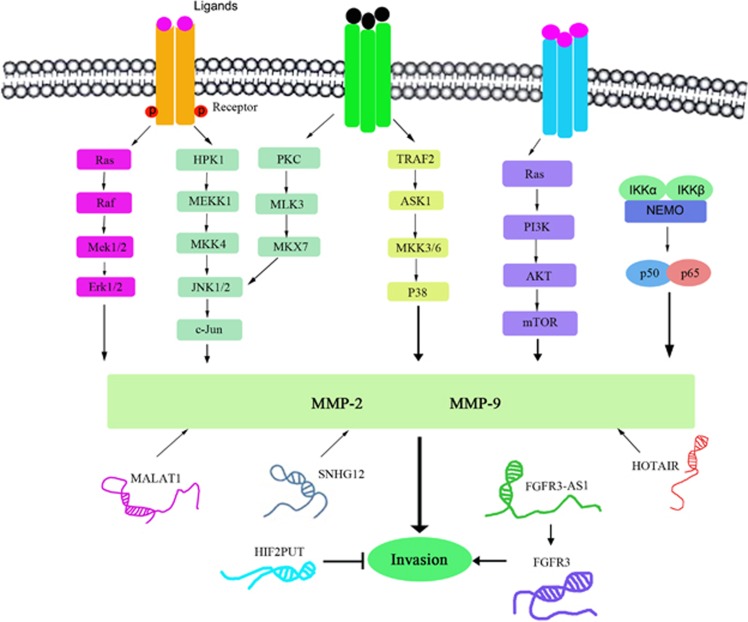
Osteosarcoma invasion and metastasis is regulated by lncRNAs, such as MALAT1, SNHG12, HOTAIR, FGFR3-AS1, and HIF2PUT. MMP-2 and MMP-9 secretion is regulated by the Erk1/2, JNK1/2, P38, PI3K/Akt, and NF-*κ*B signaling pathways. Osteosarcoma cell invasion is regulated by MMP-2 and MMP-9

Adhesion molecules, angiogenic factors, proteolytic enzymes, tumor metastasis-related factors, and metastasis suppressors are involved in migration and metastasis. MMPs are a family of proteolytic enzymes and are the key proteases involved in digesting components of the ECM and surface receptors. MMPs has an important role in tumor invasion and metastasis by degrading the ECM and basement membrane to remodel the tumor microenvironment and promote tumor angiogenesis. Conversely, MMP activity is suppressed by endogenous tissue inhibitors of metalloproteinases (TIMPs), specific MMP inhibitors. The levels of endogenous MMPs and TIMPs contribute to imbalances between MMPs and TIMPs and regulate ECM degradation and deposition. It has been reported that the levels of MMP-2 and MMP-9 secretion are elevated in several types of human cancers and that these elevations are associated with a poor prognosis.^99^ During osteosarcoma cell invasion and migration, several lncRNAs reportedly promote or inhibit cell proliferation and invasion by regulating MMP-2 and MMP-9 secretion.^100^ Osteosarcoma cell invasion and metastasis and the lncRNAs associated with these processes are shown in Figure 4.

The HOX antisense intergenic RNA (HOTAIR), a well-known lncRNA, is involved in the pathogenesis and progression of multiple tumors. HOTAIR is commonly overexpressed in osteosarcoma, and its knockdown significantly inhibits cellular proliferation and invasion by decreasing MMP-2 and MMP-9 section in osteosarcoma cells. Meanwhile, high HOTAIR expression levels are significantly associated with advanced tumor stages, high histological grades, and poor prognoses. Thus, HOTAIR may be an important target in the treatment of human osteosarcoma.^101^ It has been reported that the small nucleolar RNA host gene 12 (SNHG12) promotes cell proliferation and migration by upregulating angiomotin (AMOT) gene expression in human osteosarcoma cells.^102^ In particular, tissue samples from primary osteosarcomas (*n*=20) and adjacent normal tissues (*n*=20), as well as samples from the osteosarcoma cell lines SAOS-2, MG63, and U2OS and the human osteoblast cell line hFOB (OB3), were studied using quantitative real-time polymerase chain reaction to detect SNHG12 expression. They found that SNHG12 mRNA expression was upregulated in osteosarcoma tissues and cell lines compared with normal tissues and cells and that SNHG12 knockdown suppressed cell proliferation and migration but did not affect cell apoptosis. These findings suggest that SNHG12 lncRNA promotes cell proliferation and migration by upregulating AMOT gene expression in osteosarcoma cells *in vivo* and *in vitro* and are consistent with the findings of previous studies involving human gastric cancer patients, which showed that upregulation of SNHG15 lncRNA expression promotes cell proliferation and invasion by regulating MMP-2/MMP-9 expression.^103^ Mammalian genomes encode numerous natural antisense transcripts that are at least partially complementary to their sense transcripts. FGFR3 antisense transcript 1 (FGFR3-AS1) increased FGFR3 mRNA stability and upregulated FGFR3 expression via antisense pairing with FGFR3 3′-UTR. Increased FGFR3-AS1 expression was correlated with large tumor size, advanced Enneking stage, metastasis and poor survival. FGFR3-AS1 knockdown inhibited xenograft tumor growth of osteosarcoma cells *in vitro* and *in vivo*. Therefore, lncRNA FGFR3-AS1 promoted osteosarcoma growth by regulating its natural antisense transcript FGFR3.^104^

## LncRNA and Osteosarcoma Cell Proliferation

Cancer occurrence is characterized by uncontrolled cell cycle activity, including uncontrolled DNA replication and parental cell division.^105^ Imbalances between programmed cell death and cell proliferation contribute to the development of various cancers. Both oncogene activation and tumor suppressor gene inactivation lead to cancer occurrence and development.^106, 107^ In osteosarcoma, lncRNAs also exhibit oncogenic properties or act as tumor suppressors to control osteosarcoma progression by regulating cell cycle progression or cell apoptosis to regulate cell proliferation or migration. Antidifferentiation noncoding RNA (ANCR) is a newly identified oncogenic lncRNA that has an important role in the maintenance of cell undifferentiation. ANCR knockdown significantly inhibited U2OS and SAOS cell proliferation and U2OS cell colony formation and arrested the U2OS cell cycle at the G0/G1 phase. Moreover, ANCR regulated and controlled cell cycles by regulating the endogenous levels of cell cycle-related proteins, including p21, CDK2, and CDK4.^108^ The levels of taurine-upregulated gene 1 (TUG1) and one of its transcript variants (n377360) were significantly higher in osteosarcoma tissues compared with that in matched non-tumorous tissues. Consistent with this finding, TUG1 and n377360 suppression by siRNA significantly impaired osteosarcoma cell proliferation potential and promoted osteosarcoma cell apoptosis.^109^

Tumor suppressor lncRNAs are involved in regulating human osteosarcoma. The levels of hypoxia-inducible factor-2*α* (HIF2*α*) promoter upstream transcript (HIF2PUT), a novel lncRNA, were assessed via quantitative polymerase chain reaction in 17 osteosarcoma tissue specimens, and the data demonstrated that HIF2PUT functions as an osteosarcoma stem cell inhibitor *in vitro* partly by controlling HIF2*α* expression. HIF2PUT overexpression markedly inhibited cell proliferation and migration, decreased the percentage of CD133-expressing cells, and impaired the osteosarcoma stem sphere-forming ability of MG63 cells.^110^ It has been reported that the HIF2PUT expression levels were positively correlated with HIF2*α* expression in osteosarcoma tissues. However, HIF2PUT overexpression obviously suppressed cell proliferation and migration, decreased the percentage of CD133-expressing cells, and impaired the osteosarcoma stem sphere-forming ability of MG63 cells. However, HIF2PUT knockdown had the opposite effect. Tumor suppressor candidate 7 (TUSC7) is a potential tumor suppressor that has been shown to inhibit cell proliferation in osteosarcoma. Cong *et al.*^111^ reported that TUSC7 expression was significantly downregulated in osteosarcoma tissues compared with paired non-tumor tissues. Low TUSC7 expression is associated with poor survival (HR=0.313, 95% confidence interval (CI) 0.092–0.867) in osteosarcoma patients. Loss of TUSC7 copy number is also associated with a poor prognosis (HR=3.994, 95% CI: 1.147–13.91) in osteosarcoma patients. The author of the above study used two osteosarcoma cell lines, HOS and MG63, to investigate the biological function of TUSC7. Silencing TUSC7 increased osteosarcoma cell proliferation ability and colony formation ability. The cell cycle was not affected by TUSC7 silencing; however, the percentage of apoptotic cells decreased, and the expression levels of several proapoptotic proteins were downregulated. Importantly, xenograft tumor models were established in nude mice using MG63 cells. Silencing TUSC7 significantly promoted tumor growth *in vivo* in treated mice compared with negative-control mice. Thus, TUSC7 may be a tumor suppressor in osteosarcoma. Similarly, Wang *et al.*^112^ determined that TUSC7 is a potential biomarker for NSCLC prognosis and that TUSC7 dysregulation has an important role in NSCLC progression. In their studies, they found that the expression levels of TUSC7 were lower in NSCLC tissues and lung cancer cells compared with that in normal tissues and cells. Lower TUSC7 expression levels in NSCLC tissues were associated with larger tumor sizes and higher TNM stages. Patients with lower TUSC7 expression levels exhibited worse overall survival compared with patients with high TUSC7 expression levels. Univariate and multivariate analyses suggested that low TUSC7 expression was an independent prognostic indicator of a poor prognosis in NSCLC patients. Moreover, TUSC7 upregulation inhibited lung cancer cell proliferation *in vitro*.

## LncRNA and Osteosarcoma Prognosis

Genetic variants of HOTAIR lncRNA contribute to the risk of osteosarcoma. A two-stage, case–control study involving 900 OS patients and 900 controls was performed to evaluate the associations between HOTAIR lncRNA genetic variants and OS risk in the Chinese population, the results of which demonstrated that the C allele of rs7958904 was associated with a significantly decreased OS risk compared with the G allele (OR: 0.77; 95% CI: 0.67–0.90; *P*=6.77x10^−4^), suggesting that patients with the rs7958904 CC genotype had significantly lower HOTAIR RNA levels compared with patients with other genotypes, as well as a lower OS risk.^113^ Ma *et al.*^114^ found that TUG1 was significantly overexpressed in osteosarcoma tissues compared with matched adjacent normal tissues (*P*<0.01). Moreover, TUG1 levels were strongly correlated with disease status and tumor size, postoperative chemotherapy, and Enneking surgical stage. Furthermore, TUG1 upregulation was strongly correlated with a poor prognosis and was an independent prognostic indicator for overall survival (HR=2.78; 95% CI: 1.29–6.00; *P*=0.009) and progression-free survival (HR=1.81; 95% CI=1.01–3.54; *P*=0.037). HOTTIP was overexpressed in OS tissues and was correlated with advanced clinical stage and distant metastasis. High HOTTIP expression levels were associated with poor overall survival in OS patients. Moreover, HOTTIP expression was an independent prognostic factor for overall survival in OS patients and may represent a novel prognostic marker and therapeutic target in OS patients.^115^ Liu *et al.*^116^ demonstrated that MEG3 lncRNA levels were clearly lower in osteosarcoma tissues compared with that in adjacent non-tumor tissues. Patients with low MEG3 lncRNA expression levels exhibited shorter overall survival compared with patients with high expression levels (log-rank test, *P*<0.05). Furthermore, decreased MEG3 lncRNA expression, advanced clinical stage, and distant metastasis were all independent predictors of shorter overall survival in osteosarcoma patients.

## lncRNA and Chemotherapeutic Drug Resistance in Osteosarcoma

Surgery, radiotherapy, and chemotherapy are the three main treatments for cancer. In particular, chemotherapy has an important role in cancer therapy. However, chemotherapeutic drug resistance is the largest obstacle limiting the success of cancer therapy. Large numbers of studies have focused on chemotherapy drug resistance in human osteosarcoma, but the mechanism underlying this resistance remains to be elucidated. In osteosarcoma, chemotherapy drug efficacy is usually limited by acquired resistance to specific drugs, such as doxorubicin and cisplatin. Zhu *et al.*^117^ studied three sets of doxorubicin-resistant MG63/DXR cells and their paired parental MG63 cells and identified 3465 lncRNAs (1761 up and 1704 down) and 3278 mRNAs (1607 up and 1671 down) that were aberrantly expressed in MG63/DXR cells (fold change >2.0, *P*<0.05 and FDR<0.05). Moreover, an lncRNA-mRNA coexpression network identified lncRNAs, including ENST00000563280 and NR-036444, that interact with genes such as *ABCB1*, *HIF1A*, and *FOXC2* and may have an important role in doxorubicin resistance in OS. Several lncRNAs have been found to serve as biomarkers predicting the chemoresponses and prognoses of osteosarcoma patients, including ENST00000563280, whose expression level was significantly increased in the tissue specimens of OS patients with poor chemoresponses compared with those with good chemoresponses.

## Conclusions and Perspectives

Previous studies have reported that lncRNAs regulate the transcription, stability and translation of protein-coding genes in the mammalian genome, play important roles in regulating protein-coding genes at the transcriptional and post-transcriptional levels, and participate in important biological processes, including cell differentiation, development and human diseases.^118, 119, 120^ Human genome studies have shown that ∼18% of protein-coding genes that produce lncRNAs (10/57) are related to cancer, whereas only 9% of all human protein-coding genes (2147/23621) are related to cancer (chi-square test, *P*-value: 0.047; hypergeometric probability *P*-value = 0.018), clearly demonstrating that genes implicated in cancer development have a greater tendency to produce lncRNAs.^121^ In this review, we have summarized how lncRNAs regulate cell proliferation, invasion and chemotherapeutic drug resistance in human osteosarcoma patients and osteosarcoma cells. We have also summarized the roles of lncRNAs as prognostic biomarkers in osteosarcoma therapy. Finding promising therapeutic targets for the treatment for human osteosarcoma, especially chemotherapeutic drug-resistant osteosarcoma, will be beneficial for patients. However, several questions regarding the involvement of lncRNAs in osteosarcoma remain unexplored and unresolved.

1. To date, limited studies regarding the involvement of lncRNAs in human osteosarcoma have been published. Although several lncRNAs are known to exert tumor-promoting or tumor-suppressing effects in osteosarcoma species and cancer cell lines, the exact molecular mechanisms underlying these effects remain unclear. Thus, additional investigations are required to elucidate the molecular mechanisms underlying human osteosarcoma progression, metastasis and drug resistance.
2. One lncRNA may be involved in several different signaling pathways associated with cancer development and may have more than one target associated with osteosarcoma proliferation and metastasis. For instance, MALAT1 plays an important role in the PI3K/AKT and RhoA/ROCKs signaling pathways. However, understanding the connections between these signaling pathways, as well as determining whether one of them plays a major role in osteosarcoma development and progression, warrants further study.
3. lncRNAs usually have short half-lives and exhibit low transcript abundance. They need to be transiently expressed *in vitro*. Additionally, it is necessary to determine how the secondary and tertiary structures of lncRNAs interact with specific protein targets.
4. lncRNAs may represent novel therapeutic targets, which are critical for developing novel strategies for the early diagnosis and treatment of human osteosarcoma. The potential clinical applications of miRNAs warrant investigation.

## Figures and Tables

**Table 1:** Overview of lncRNA in osteosarcoma

**Name**	**Functions**	**Up/downregulation**	**Regulation mechanism**	**Reference**
H19	Oncogene	+	lncRNA H19 was one of the candidates with the highest relevance in cancer development H19 is induced by Yap1 overexpression in the mouse osteosarcoma tissues and primary osteosarcoma cells.	^98^
MALAT1	Oncogene	+	MALAT1 might be an oncogenic lncRNA that promoted proliferation and metastasis of osteosarcoma by PI3K/AKT signaling pathway in osteosarcoma cell lines and clinical tumor samples Knockdown of the putative Myc-6 target MALAT1 significantly impaired cell growth of MG63 cells, suggesting that Myc-6 might exert its tumor-suppressive ability in part through the specific downregulation of MALAT1.	^94, 95^^,97^
ANCR	Oncogene	+	ANCR worked as an oncogenic lncRNA that promoted proliferation of osteosarcoma by regulalting the cell cycle progression The cell cycle of U2OS cells was arrested at the G0/G1 phase and the expression level of p21 was increased and CDK2 was decreased in ANCR knockdown cells..	^108^
HOTAIR	Oncogene	+	Overexpression of HOTAIR promoted tumor growth and metastasis by increasing the secretion of MMP-2 and MMP-9 in human osteosarcoma High levels of HOTAIR were correlated with advanced tumor stage and poor prognosis.	^101^
PACER	Oncogene	+	PACER was overexpressed in clinical osteosarcoma tissues and cell lines. Its overexpression promoted proliferation and metastasis of osteosarcoma cells by activating *COX*-2 gene in an NF-*κ*B-dependent manner.	^92^
SNHG12	Oncogene	+	SNHG12 expression was upregulated in tissue samples from primary osteosarcoma and adjacent normal tissues, the osteosarcoma cell lines, SAOS-2, MG63, U-2 OS, and the human osteoblast cell line hFOB (OB3), by upregulating AMOT gene expression.	^102^
FGFR3-AS1	Oncogene	+	The expression of FGFR3-AS1 and FGFR3 was positively correlated in osteosarcoma tissues FGFR3-AS1 promoted osteosarcoma growth through regulating its natural antisense transcript FGFR3.	^104^
ENST00000563280, NR-036444	Oncogene	+	The lncRNA had a critical role in doxorubicin resistance of OS by interacting with important genes such as *ABCB1*, *HIF1A*, and *FOXC2*.	^117^
ENST00000563280	Oncogene	+	The lncRNA was significantly increased in specimens of OS patients with a poor chemoresponse compared with those with a good chemoresponse The patients with lower expression of ENSt00000563280 may survived longer compared with those of higher expression.	^117^
TUG1	Oncogene	+	TUG1 was overexpressed in patients with osteosarcoma TUG1 was positively correlated with disease status It may serve as a molecular indicator in maintaining surveillance and forecasting prognosis.	^114^
HOTTIP	Oncogene	+	HOTTIP was overexpressed in OS tissues HOTTIP was correlated with advanced clinical stage and distant metastasis OS patients with high HOTTIP expression level had poorer overall survival compared with those with low HOTTIP expression It could be a novel prognostic marker and potential therapeutic target in OS patients.	^115^
MEG3	Tumor suppressor	−	Liu *et al.* found that the expression of lncRNA MEG3 was associated with clinical stage and distant metastasis The patients with low lncRNA MEG3 expression had a shorter overall survival MEG3 was an independent predictor to overall survival of osteosarcoma patients.	^116^
HIF2PUT	Suppressor gene	−	HIF2PUT is a novel regulatory factor of osteosarcoma stem cells HIF2PUT exerted its function partly by controlling HIF2*α* expression.	^110^
TUSC7	Tumor suppressor	−	TUSC7 was significantly downregulated in osteosarcoma tissues TUSC7 regulated the cell proliferation ability and colony formation ability involved in regulating the expression of proapoptotic proteins.	^111^

Abbreviations: AMOT, angiomotin; ANCR, antidifferentiation noncoding RNA; FGFR3-AS1, FGFR3 antisense transcript 1; HIF2*α*, hypoxia-inducible factor-2*α*; HIF2PUT, HIF2*α* promoter upstream transcript; HOTAIR, HOX antisense intergenic RNA; HOTTIP, HOXA transcript at the distal tip; lncRNA, long noncoding RNA; MALAT1, metastasis-associated lung adenocarcinoma transcript 1; MEG3, maternally expressed gene 3; MMP, matrix metallopeptidase; NF-*κ*B, nuclear factor-*κ*B; PACER, P50-associated COX-2 extragenic RNA; SNHG12, small nucleolar RNA host gene 12; TUG1, taurine-upregulated gene 1; TUSC7, tumor suppressor candidate 7; yap1, yes-associated protein 1

## Abbreviations

siRNA: small interfering RNA
microRNA: micro RNA
lncRNA: long noncoding RNA
Hh signaling pathway: hedgehog signaling pathway
BMP: bone morphogenetic protein
PI3K: phosphatidylinositol 3-kinase
PACER: P50-associated COX-2 extragenic RNA
MALAT1: metastasis-associated lung adenocarcinoma transcript 1
MMP-9: matrix metallopeptidase 9
Yap1: yes-associated protein 1
ECM: extracellular matrix
TIMP: inhibitors of metalloproteinase
HOTAIR: HOX antisense intergenic RNA
SNHG12: small nucleolar RNA host gene 12
AMOT: angiomotin
FGFR3-AS1: FGFR3 antisense transcript 1
HIF2PUT: hypoxia-inducible factor-2*α* (HIF2*α*) promoter upstream transcript
TUSC7: tumor suppressor candidate 7
